# ChatGPT’s ability to generate realistic experimental images poses a new challenge to academic integrity

**DOI:** 10.1186/s13045-024-01543-8

**Published:** 2024-05-01

**Authors:** Lingxuan Zhu, Yancheng Lai, Weiming Mou, Haoran Zhang, Anqi Lin, Chang Qi, Tao Yang, Liling Xu, Jian Zhang, Peng Luo

**Affiliations:** 1 Department of Oncology, Zhujiang Hospital, Southern Medical University, 253 Industrial Avenue, 510282 Guangzhou, Guangdong China; 2 Department of Urology, Shanghai General Hospital, Shanghai Jiao Tong University School of Medicine, Shanghai, China; 3 Institute of Logic and Computation, TU Wien, Wien, Austria; 4 Department of Medical Oncology, National Clinical Research Center for Cancer /Cancer Hospital, National Cancer Center, Chinese Academy of Medical Sciences and Peking Union Medical College, Beijing, China

**Keywords:** Academic integrity, ChatGPT, DALL-E, Large language model, Experimental images, Western Blot, Artificial intelligence

## Abstract

The rapid advancements in large language models (LLMs) such as ChatGPT have raised concerns about their potential impact on academic integrity. While initial concerns focused on ChatGPT’s writing capabilities, recent updates have integrated DALL-E 3’s image generation features, extending the risks to visual evidence in biomedical research. Our tests revealed ChatGPT’s nearly barrier-free image generation feature can be used to generate experimental result images, such as blood smears, Western Blot, immunofluorescence and so on. Although the current ability of ChatGPT to generate experimental images is limited, the risk of misuse is evident. This development underscores the need for immediate action. We suggest that AI providers restrict the generation of experimental image, develop tools to detect AI-generated images, and consider adding “invisible watermarks” to the generated images. By implementing these measures, we can better ensure the responsible use of AI technology in academic research and maintain the integrity of scientific evidence.

**Supplementary Information:**

The online version contains supplementary material available at 10.1186/s13045-024-01543-8.

## To the editor

The impacts of large language models (LLMs) such as ChatGPT on academic integrity have received increasing attention. Initial concerns focused on ChatGPT’s writing abilities being exploited for academic writing, leading several publishers to ban ChatGPT as an author [1, 2]. In addition to writing articles, a recent study found ChatGPT can generate fake but realistic research datasets from scratch to support a predetermined conclusion [3]. Furthermore, in a recent update, ChatGPT integrated the DALL-E 3’s image generation capabilities, allowing users to easily create various high-quality images with simple text prompts [4]. This could extend concerns about ChatGPT’s impacts on academic integrity from text to images, posing an entirely new challenge.

Images serve as crucial evidence supporting conclusions in biomedical research papers but are also susceptible to manipulation. For instance, Western Blot (WB) is an experiment used to detect the concentration of a target protein in a sample. Researchers’ judgement of protein concentration is entirely based on the intensity of the corresponding bands in the image. Unfortunately, the reliance on visual evidence has opened the door to falsify data through image manipulation. The earliest methods involved techniques like rotation, splicing, and retouching, but careful inspection could detect traces of manipulation [5]. With the exposure of paper mills, some reports suggest they use an artificial intelligence (AI) technology called Generative Adversarial Networks (GAN) to generate fabricated WB results that align with desired outcomes [6]. Qi et al. developed a GAN model to generate WB images and found that the synthetic fake images could not be identified by human observers [7]. Nevertheless, the GAN technique has a high barrier and not everyone can use it to generate experimental images. However, ChatGPT’s new image generation feature changes this. Alarmingly, our simple tests revealed that ChatGPT’s nearly barrier-free image generation feature can be used to generate realistic experimental result images.

**Fig. 1:**
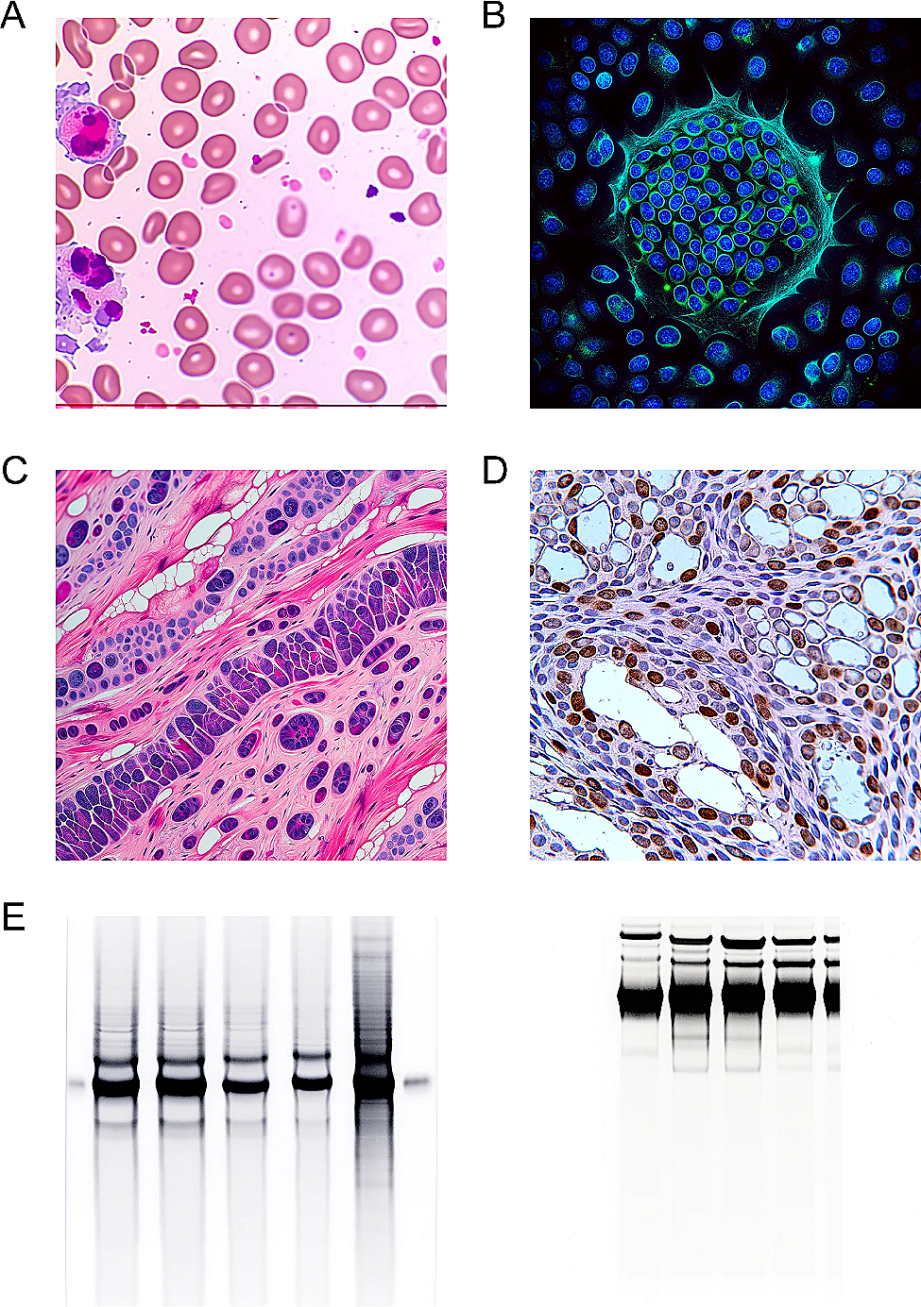
Realistic experimental images generated using ChatGPT. (**A**) blood smears. (**B**) immunofluorescence staining. (**C**) hematoxylin and eosin (H&E) staining. (**D**) immunohistochemistry. (**E**) western blot images

We tried to use this new feature to request ChatGPT to generate realistic blood smears, immunofluorescence staining, hematoxylin and eosin (H&E) staining, immunohistochemistry and WB images (Fig. 1, see Supplementary Material for the prompt used). The results are striking, and some of the images generated by ChatGPT have been very close to those obtained from real experimental results, especially the blood smears and the immunofluorescence images, which could probably fool some people who are less experienced in biomedical experiments.

Although the current ability of ChatGPT to generate experimental images is limited, our simple tests have demonstrated the significant risks of misuse in generating images. Combined with existing research findings, ChatGPT theoretically has the potential to generate entire academic papers from scratch, including text, raw data, and experiment result images. While images generated by ChatGPT currently are not as realistic as those generated by GANs, the low barrier to use and rapid technical improvements mean the generated images will likely be more realistic in future. This risk is not limited to ChatGPT, but also exists in all popular LLMs that can generate images. In addition to generating complete experimental images from scratch, AI technology could also be misused to partially or locally modify real images obtained from experiments. For example, researchers might use AI tools to selectively enhance or weaken the intensity of specific bands in Western Blot results to support predetermined conclusions. This could be more difficult to detect as the final images are a hybrid of real experimental images and AI-generated content. We believe it is imperative to promptly acknowledge this potential harm and take immediate action, urging AI technology providers to restrict the generation of experimental images. In addition, tools should be developed to help us determine whether images are generated by AI systems, similar to the tools used to detect whether text is generated by ChatGPT [8]. Moreover, AI technology providers should consider adding “invisible watermarks” to the generated images, which cannot be recognized by the naked eye but can be detected by specific tools. This can help us more accurately identify whether the images are AI-generated [9]. By implementing these measures, we can better mitigate the risks associated with AI-generated images and ensure a more responsible use of this technology.

### Electronic supplementary material

Below is the link to the electronic supplementary material.

Supplementary Material 1

## Data Availability

No datasets were generated or analysed during the current study.

## Abbreviations

AI: Artificial Intelligence
GAN: Generative Adversarial Networks
H&E: Hematoxylin and Eosin
LLMs: Large Language Models
WB: Western Blot
