# Supplementary material for: ChatGPT’s ability to generate realistic experimental images poses a new challenge to academic integrity
Source: J Hematol Oncol. 2024 May 1;17:27. doi: 10.1186/s13045-024-01543-8 (PMC11064365; doi:10.1186/s13045-024-01543-8)
Supplement: Supplementary file 1 — Supplementary Material 1 [file 13045_2024_1543_MOESM1_ESM.docx]

**Supplementary Materials.** Prompts used for this article.

|  | Prompt |
| --- | --- |
| Blood Smears | Create an intricate and authentic 2D representation of a blood smear as seen through an optical microscope, emphasizing that the view is planar with minimal depth of field. The visual field should primarily feature red blood cells in a uniform color and transparency, accurately reflecting the real-life proportions and uniform appearance of these cells in human blood. These red blood cells should be depicted as biconcave disks with a consistent soft pink hue, occupying the majority of the image to illustrate their abundance in the bloodstream. Sparse among them, include a limited variety of white blood cells to avoid an unrealistic representation; such as a few neutrophils with their multi-lobed nuclei and granular appearance, along with some lymphocytes with large, spherical, dark purple nuclei that dominate the cell's volume. A single, prominent white blood cell, indicative of a mature neutrophil with a segmented nucleus and distinctive purple staining, should be evident. The pale pink background should be reminiscent of a stained glass slide, indicative of the medium of the blood smear. All cellular details must be presented with sharp, clear boundaries and in complete focus to emulate the high-resolution clarity of an optical microscope. The colors should be vivid and consistent with the appearance of a Wright's stained blood smear. This image is to be a factual and precise visual representation, capturing the planar, detail-rich view of a microscopic field, staying true to the natural cell ratio and uniformity of red blood cells seen in a blood sample under an optical microscope. |
| Immunofluorescence Staining | Create a highly detailed image showing the results of a realistic immunofluorescence assay. The image should depict a multitude of cells against a dark background. Each cell should have a distinct nucleus stained in bright blue, and the cytoplasm should be visible in a contrasting green fluorescence. The green should show specific protein localization, appearing as dots or specific patterns within the cells. There should be no other colors present in the image. The overall appearance should resemble a microscopic view, with cells in various stages of the cell cycle, some clustered together and some isolated. The image should capture the intricate details of cellular structure and the specificity of immunofluorescence staining, evoking the sense of a scientific image taken under a high-powered fluorescence microscope. |
| Hematoxylin and  Eosin (H&E) staining | Create a detailed and realistic digital painting of a microscopy image showing the results of an immunohistochemistry staining experiment. The image should mimic the appearance of hematoxylin and eosin (H&E) staining. It should feature a variety of pink and purple hues that represent the different intensities of staining, indicating the presence of various cellular components. The pink areas should represent lighter staining, typically of muscle and connective tissues, while the darker purples should indicate nuclei and dense areas of cells. The tissue should be structured with clear demarcations between cells and the extracellular matrix, showing a typical pattern of organized tissue architecture. The slide should have a fibrous texture, characteristic of connective tissue, with visible cells that have prominent purple nuclei surrounded by a lighter pink cytoplasm. The overall composition should resemble a close-up, with enough detail to give the impression of looking through a microscope at a 400x magnification. The image should be rich in detail to reflect the complexity of tissue structure, with subtle variations in staining intensity that suggest depth and the three-dimensional arrangement of cells within the tissue matrix. |
| Immunohistochemistry | Create an image that depicting an authentic Immunohistochemistry (IHC) experiment result. The image should display a magnified view of a tissue section, with evident cellular structures. The cells are to be in a complex, intertwining pattern typical of epithelial tissue. Each cell is to have a distinct, round to oval-shaped nucleus stained dark blue, surrounded by lighter cytoplasm. Brown staining, indicative of a positive IHC reaction, should be visible on the cell membranes and sometimes within the cytoplasm, signifying specific antigen-antibody interactions. The color scheme should consist of various shades of brown for the staining, with blue nuclei and pinkish cytoplasm. The tissue should be depicted with detailed accuracy, showing layers of cells in a two-dimensional plane, closely reflecting the histological structure found in a genuine IHC analysis. The image should be sharp, with distinct borders between the different staining regions, and should accurately represent the histopathological features of the tissue as it would appear under a microscope in a real IHC test. |
| Western Blot images | Create a realistic and detailed image of a Western Blot assay result with a stark white background and crisp black bands. The image should feature a series of six vertical lanes, each with a sequence of horizontal bands. These bands should vary in intensity to represent the presence of proteins at different molecular weights. The bands must be sharp, straight, and evenly spaced, indicative of a precise scientific measurement rather than artistic interpretation. They should be uniform in width and run parallel to each other, with no smudging or bleeding, simulating a high-quality Western Blot experiment. The darkest bands should be at the bottom of each lane, gradually lightening towards the top. This image must embody the clarity and distinct separation of bands typical in a real-life Western Blot, reflecting the common laboratory practice of protein separation and identification. |
